# Body Weight Changes During Pandemic-Related Shelter-in-Place in a Longitudinal Cohort Study

**DOI:** 10.1001/jamanetworkopen.2021.2536

**Published:** 2021-03-22

**Authors:** Anthony L. Lin, Eric Vittinghoff, Jeffrey E. Olgin, Mark J. Pletcher, Gregory M. Marcus

**Affiliations:** 1 Department of Medicine, University of California, San Francisco; 2 Department of Epidemiology and Biostatistics, University of California, San Francisco; 3 Division of Cardiology, Department of Medicine, University of California, San Francisco

## Abstract

This cohort study investigates whether the shelter-in-place orders in the US during the early weeks of the COVID-19 pandemic were associated with changes in body weight among adults.

## Introduction

As of January 22, 2021, there were more than 98 million confirmed cases of severe acute respiratory syndrome coronavirus 2 (SARS-CoV-2), more than 24 million of which are attributed to the US alone.^1^ Recent surges in SARS-CoV-2 and the threat of a second wave have prompted many states to reconsider reopening timelines. During the initial US surge, 45 out of 50 state governments issued shelter-in-place (SIP) orders from March 19, 2020, to April 6, 2020, to slow disease transmission.^2^ The initial SIP coincided with an observed decrease in daily step counts,^3^ likely reflective of changes in physical activity and patterns of daily living, as well as concurrent self-reported increases in snacking and overeating.^4^ We therefore sought to investigate ambulatory weight changes of a longitudinal cohort during initial SIP orders to better understand the possible downstream health implications of prolonged SIP.

## Methods

This cohort study was approved by the University of California, San Francisco (UCSF) institutional review board, and informed consent was obtained from all participants. This study followed the Strengthening the Reporting of Observational Studies in Epidemiology (STROBE) reporting guideline for cohort studies.

We performed a longitudinal analysis of data obtained from February 1 to June 1, 2020, from participants in the Health eHeart Study who volunteered to report weight measurements from their Bluetooth-connected smart scale (Fitbit [Fitbit Inc] or iHealth [iHealth Labs Inc]). Additional study design and study population selection details are in the eAppendix in the Supplement.

Demographic characteristics and medical conditions were obtained via online surveys. Race and ethnicity of participants were assessed via self-report at time of enrollment. Weight change before and after SIP was studied via a linear mixed-effects model with a spline point at the day SIP orders were issued for each state. Random intercepts, random slopes, and first-order autoregressive residuals were used to track within-group changes for each participant. A 2-tailed *P* < .05 was considered statistically significant. All analyses were performed using R version 4.0.0 (R Project for Statistical Computing) from February to May 2020.

## Results

A total of 7444 weight measurements from 269 unique study participants (residing in 37 states and Washington, District of Columbia) were collected during the study period, with a mean (SD) of 28 (24) weight measurements per participant. Of 269 study participants, 130 (48.3%) were men and 207 (77.0%) were White individuals; and age data was available for 169 participants (62.8%) with a mean (SD) age of 51.9 (17.3) years. Baseline characteristics are displayed in the Table. As illustrated in the Figure, post-SIP participants experienced steady weight gain at a rate of 0.27 kg every 10 days (95% CI, 0.17 to 0.38 kg per 10 days; *P* < .001), irrespective of geographic location or comorbidities. These results translate into approximately 1.5 lb of weight gain every month (to convert kilograms to pounds, divide by 0.45).

**Table.** Demographic and Clinical Characteristics of Participants

Characteristics	Patients, No. (%)		*P* value
	Below median weight (n = 134)	Above median weight (n = 135)	
Age, mean (SD), y	50.6 (18.8)	52.0 (15.6)	NA
Sex			
Female	75 (56)	42 (31)	<.001
Male	47 (35)	83 (61)	
Not reported or unknown	12 (9)	10 (7)	.81
Race			
Black or African American	5 (4)	4 (3)	.99
White	100 (75)	107 (79)	.45
Asian	7 (5)	1 (1)	.07
Identified as 2 or more races	6 (4)	5 (4)	.99
Other	4 (3)	7 (5)	.55
Not reported or unknown	12 (9)	11 (8)	>.99
Ethnicity			
Hispanic	6 (4)	10 (7)	.45
Not reported or unknown	12 (9)	12 (9)	>.99
Geographical region			
West	51 (38)	42 (31)	.29
Midwest	21 (16)	21 (16)	>.99
Northeast	23 (17)	26 (19)	.77
South	39 (29)	46 (34)	.46
Medical comorbidities			
Hypertension	35 (26)	66 (49)	<.001
Hyperlipidemia	46 (34)	54 (40)	.40
Diabetes	8 (6)	18 (13)	.66
Coronary artery disease	9 (7)	10 (7)	>.99
Congestive heart failure	5 (4)	2 (1)	.44
Atrial fibrillation	9 (7)	20 (15)	.05
Chronic obstructive pulmonary disease	4 (3)	2 (1)	.67
Sleep apnea	9 (7)	31 (23)	<.001
History of myocardial infarction	6 (4)	6 (4)	>.99
History of stroke	3 (2)	3 (2)	>.99
Weight, mean (SD), kg	68.1 (9.1)	101.0 (15.4)	NA
Body mass index, mean (SD)^a^	24.1 (3.0)	32.9 (5.6)	NA

Abbreviation: NA, not applicable.

^a^ Body mass index is calculated as weight in kilograms divided by height in meters squared.

**Figure.**
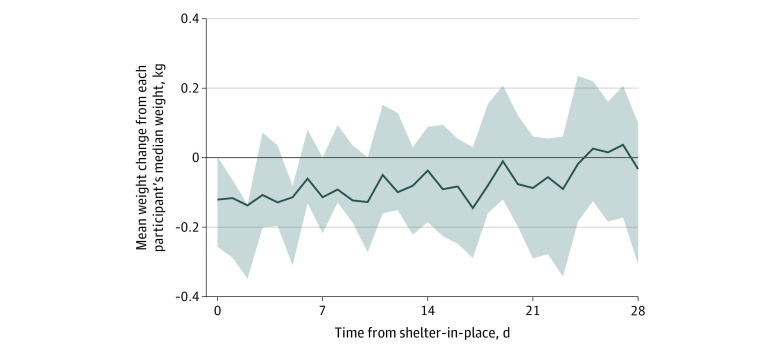
Mean Weight Change After Shelter-in-Place for the Study Population Figure data normalized as weight above or below each participant’s median weight in kilograms. Shaded areas denote the 95% CI for the mean weight of study participants after shelter-in-place.

## Discussion

Weight is a clinically relevant health outcome that is independently associated with all-cause mortality.^5^ It is also a helpful proxy for physical activity, another measurement associated with all-cause mortality.^6^ In analyzing weight trends around initial SIP, we found a significant increase in weight over the post-SIP period at a rate of roughly a pound and a half weight gain per month following SIP. Although this may not appear clinically important, prolonged effects as have occurred with the pandemic might lead to substantial weight gain.

Because of the reliance on Bluetooth-connected scales and weight measurements during SIP from participants of the Health eHeart study, reduction of overall sample size is a limitation to this study. Although idiosyncratic characteristics of those who happen to own a Bluetooth-connected scale may limit the study’s generalizability, following individuals over time to assess their objectively measured weight changes during SIP diminishes threats to internal validity.

It is important to recognize the unintended health consequences SIP can have on a population level. The detrimental health outcomes suggested by these data demonstrate a need to identify concurrent strategies to mitigate weight gain, such as encouraging healthy diets and exploring ways to enhance physical activity, as local governments consider new constraints in response to SARS-CoV-2 and potential future pandemics.

## Notes

Supplement. **eAppendix.** Supplementary Methods
