# Supplementary material for: Body Weight Changes During Pandemic-Related Shelter-in-Place in a Longitudinal Cohort Study
Source: JAMA Netw Open. 2021 Mar 22;4(3):e212536. doi: 10.1001/jamanetworkopen.2021.2536 (PMC7985720; doi:10.1001/jamanetworkopen.2021.2536)
Supplement: Supplement. — eAppendix. Supplementary Methods [file jamanetwopen-e212536-s001.pdf]

Supplemental Online Content

Lin AL, Vittinghoff E, Olgin JE, Pletcher MJ, Marcus GM. Body weight changes during pandemic-related shelter-in-place in a longitudinal cohort study. *JAMA Netw Open.* 2021;4(3):e212536. doi:10.1001/jamanetworkopen.2021.2536

eAppendix. Supplementary Methods

This supplemental material has been provided by the authors to give readers additional information about their work.

© 2021 Lin AL et al. *JAMA Network Open.*

eAppendix. Supplementary Methods

The Health eHeart Study is an internet-based longitudinal cardiovascular e-cohort launched on March 8, 2013 and is still ongoing. Enrollment of participants was done worldwide via lay press, social media, email campaigns, and word-of-mouth. All English-speaking adults aged 18 years and over with an active email address were eligible for enrollment. Enrollment was voluntary and no incentives were offered for study participation. Consent was obtained electronically through the study website. Upon enrollment, participants were prompted to complete a series of baseline questionnaires that asked about basic demographics, family history, medical comorbidities, social demographics, and health behaviors. Age was self-reported as a continuous integer that participants provided on enrollment. Self-identified sex was dichotomized as male or female. Self-identified race was categorized as Black or African American, White, Asian or Pacific Islander, or other, which gave participants an opportunity to identify as another race. Ethnicity was dichotomized as Hispanic or not. Geographic region was based on self-reported zip codes provided by participants. Medical comorbidities were ascertained via self-report and participants were asked to report diagnoses of hypertension, hyperlipidemia, diabetes, coronary artery disease, congestive heart failure, atrial fibrillation, chronic obstructive pulmonary disease, sleep apnea, a history of myocardial infarction, and a history of stroke. For any of the baseline questionnaires, participants were allowed to leave any question unanswered should they wish not to share that information.

© 2021 Lin AL et al. *JAMA Network Open.*

The study includes the capability to synchronize a Bluetooth-connected smart scales from FitBit (i.e. Fitbit Aria Air, Fitbit Aria 2, etc.) or iHealth (i.e. iHealth Lite Wireless Body Analysis Scale, etc.). Participants with smart scales could opt to voluntarily contribute their weight data to the Health eHeart Study. A total of 2,336 unique participants have contributed 577,933 weight measurements through a Bluetooth-connected smart scale since the feature was introduced.

Shelter-in-place recommendations were made in 45 out of 50 states throughout the United States from March 19, 2020 to April 6, 2020 in response to the global SARS-CoV-2 pandemic. Health eHeart participants who contributed at least 1 weight measurement before and 1 measurement after their state-specific shelter-in-place order was placed were included in this study, which reduced the study sample size to 269 unique participants.

Continuous variables were presented as means and standard deviations and compared using t-tests, while categorical variables were presented as frequencies (percentages) and compared using the chi-squared test. As participants volunteered weight measurements from their home smart scales only when performed, a weight was not recorded for all participants for every day in the study period. Therefore, a linear mixed effects model was employed to enable analysis of aggregate body weight changes during this time period. A spline point at the day shelter-in-place orders were issued for each state was used to assess for any changes in body weight immediately following state mandates promoting shelter-in-place. The use of random intercepts, random slopes, and first-order autoregressive residuals was employed to allow the model to account for variability in baseline weight and different rates of body weight change between participants during the

© 2021 Lin AL et al. *JAMA Network Open.*

study period. A two-tailed p-value of <0.05 was considered statistically significant. All analyses were done using R version 4.0.0.

© 2021 Lin AL et al. *JAMA Network Open.*
